# Decoding RNF20: an epigenetic modifier and beyond

**DOI:** 10.3389/fcell.2026.1791026

**Published:** 2026-04-02

**Authors:** Jie Ren, Weimei Ou, Rui Gao

**Affiliations:** Institute of Cardiovascular Diseases, Xiamen Cardiovascular Hospital of Xiamen University, School of medicine, Xiamen University, Xiamen, China

**Keywords:** E3 ligase, epigenetic regulation, H2Bub1, RNF20, ubiquitination

## Abstract

E3 ubiquitin ligases play essential roles in catalyzing the ubiquitination process and are involved in almost all life activities of eukaryotes. RNF20 is a really interesting new gene (RING) finger E3 ubiquitin ligase and is well-known for its role in monoubiquitination of histone H2B. Recent studies have offered new insights into how RNF20 regulates gene expression and developmental programs, and how misregulation of its activities leads to pathologies including developmental disorders and cancers. Here, we provide a current review of the physiological and pathological roles of RNF20 in embryonic development and human diseases, and outline its cellular and molecular modes of action, as well as the upstream control of RNF20 activity, thus providing insights for the molecular basis of RNF20 misregulation associated human diseases.

## 1 Introduction

Posttranslational modifications (PTMs) are covalent processing events that change the properties of a protein by proteolytic cleavage and adding a modifying group. PTMs play a key role in numerous biological processes by significantly affecting the structure and activity of proteins. Abnormal PTMs may cause changes or loss of protein functions, which are closely related to the occurrence and development of various diseases. Ubiquitination (or termed as ubiquitylation) is one of the most studied PTMs. Ubiquitination is a sequential three-step enzymatic cascade involving ubiquitin-activating enzymes (E1s), ubiquitin conjugating enzymes (E2s) and ubiquitin ligase enzymes (E3s) (Damgaard, 2021). In the first step, ubiquitin (Ub) is attached to E1 in an ATP-dependent manner. Next, the ubiquitin is transferred to a cysteine on an E2-conjugating enzyme. In the last step, the Ub is transferred from E2 to a substrate either directly from the E2, mediated by a RING (really interesting new gene) E3 Ub ligase, or first transferred to a HECT/RBR (Homologous to the E6-AP Carboxyl Terminus/RING-in-between-RING) E3 Ub ligase before being transferred to the substrate (Damgaard, 2021).

RING finger protein 20 (RNF20, also called BRE1A) is one of the RING E3 ligase, which is well-known for its role in monoubiquitination of histone H2B (H2Bub1). In recent years, there are a growing number of advances in unraveling the roles of RNF20 in multiple biology processes including embryonic development, functional maintenance in adult tissues, as well as tumor initiation and progression. In this review, we focus on the significant progresses of RNF20’s roles in physiological and pathological conditions; outline its molecular modes of action and the upstream regulations of RNF20 activity, thus providing insights for the molecular basis of RNF20 in human health and diseases.

## 2 Structure of RNF20

**FIGURE 1:**
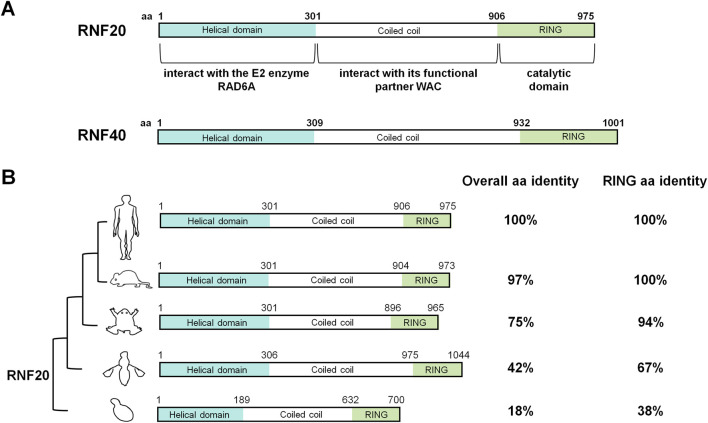
RNF20 domain structure feature and conservation in genetic models. **(A)** Schematic diagram of human RNF20 and RNF40 protein. Human RNF20 and RNF40 are composed of 975 and 1001 amino acids (aa), respectively, consisting of an N-terminal helical domain, a coiled-coil region and a C-terminal RING domain. **(B)** Phylogenetic analysis of RNF20 protein and protein domain architecture in different genetic models. Amino acid identity of overall sequence and RING domain between human RNF20 and RNF20 proteins from other species in National Center for Biotechnology Information (NCBI) database are indicated. RNF20 Protein sequence reference number in NCBI: *Homo sapiens* (NP_062538.5); *Mus musculus* (NP_001156735.1); *Xenopus tropicalis* (XP_002941911.2); *Drosophila melanogaster* (NP_647989.2); *Saccharomyces cerevisiae* (NP_010209.1).

Human *RNF20* gene locates on chromosome 9 and comprises 20 exons, encoding a protein of 975 amino acids. RNF20 protein contains an N-terminal helical domain and a C-terminal RING domain, which are connected by a long coiled-coil region (Figure 1) (Deng et al., 2023; Onishi et al., 2024). The N-terminal helical domain is responsible for the interactions of RNF20 with its E2 enzyme RAD6A (Onishi et al., 2024). The coiled-coil region of RNF20 has been shown to interact with its functional partner, *WW* domain-containing adaptor with coiled-coil (WAC) (Zhang and Yu, 2011). The RING domain of RNF20 is its catalytic domain which recruits the E2 enzyme and ubiquitin for site-specific ubiquitination (Onishi et al., 2024). RNF20 is an evolutionarily conserved factor from yeast to humans, especially apparent in their RING domains (Figure 1) (Hwang et al., 2003; Wang et al., 2017).

RNF20 is reported to form a stable heterodimer with RNF40 (also called BRE1B) and knockdown of one of them reduced the level of the other (Kim et al., 2009; Moyal et al., 2011; Foglizzo et al., 2016). RNF40 is a paralog of RNF20, which is highly similar with RNF20 in protein sequence with 58% overall amino acids (aa) identity and 87% RING domain sequence identity (Onishi et al., 2024). RNF40 locates on chromosome 16 and comprises 21 exons, encoding a polypeptide of 1001 amino acids. However, RNF20 and RNF40 play asymmetrical functions in catalysis, even though their sequences are similar. In the process of H2B monoubiquitination, RNF20, but not RNF40, is the one which functions as a catalytic subunit responsible for recruiting the E2 enzyme RAD6A and ubiquitin (Onishi et al., 2024).

Importantly, as an E3 Ub ligase of histone H2B, how RNF20 Ub machinery is recruited to the chromatin was a question. Recently, this question has been solved by structure studies from multiple groups that the RING domain of RNF20 directly interacts with nucleosomal DNA since it has positively charged molecular surfaces, thus can bind to the negatively charged acidic patch and DNA phosphate groups in the nucleosome through electrostatic interactions (Deng et al., 2023; Zhao et al., 2023; Onishi et al., 2024).

## 3 Control of RNF20 activity

**FIGURE 2:**
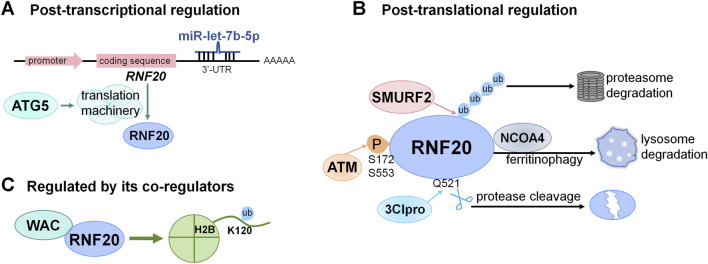
Cellular control of RNF20 activity at different levels. **(A)** Schematic diagram of post-transcriptional regulations on RNF20 by miR-let-7b-5p or ATG5. **(B)** Model of post-translational modifications or upstream regulations targeting RNF20. **(C)** Control of RNF20 activity by its co-regulators such as WAC. See details in the context.

RNF20 activity can be controlled at various levels (Figure 2). Firstly, RNF20 has been shown to be regulated at the post-transcriptional level which affects RNF20 protein expression level (Figure 2A). One of the regulators is microRNA, which interacts with the target mRNA to induce translational repression and mRNA degradation. Pancreatic cancer-derived exosomal microRNA miR-let-7b-5p has been shown to target 3′untranslated region (3′-UTR) of RNF20 in C2C12 myotube cells and downregulates its expression (Wang et al., 2023). Moreover, another post-transcriptional regulator of RNF20 is autophagy-related protein ATG5, which has been reported to regulate RNF20 by promoting its translation (Huang et al., 2017). In details, ATG5 interacts with eukaryotic initiation factor eIF4A and facilitates its association with eIF4G to form the translation initiation complex (Huang et al., 2017). In parallel, ATG5 also controls the activity of the protein translation machinery by protects the phosphorylation of eIF4B and eIF4G, thus facilitating the translation efficiency of RNF20 mRNA (Huang et al., 2017).

Secondly, emerging evidence has demonstrated that RNF20 is also finely regulated at post-translational level (Figure 2B). One critical regulatory way is ubiquitination. Smad ubiquitin regulatory factor 2 (Smurf2), which is required for maintaining the histone modification pattern over the general chromatin landscape and for regulating the acute DNA damage response at sites of DNA double-stranded breaks by fine tuning the amount of RNF20, is reported to be the E3 ubiquitin ligase of RNF20 and targets RNF20 for proteasomal degradation by polyubiquitination (Blank et al., 2012). Another known regulatory way on RNF20 is ferritinophagy. On ferritinophagy activation, the ferritinophagy-specific cargo receptor nuclear receptor coactivator 4 (NCOA4) binds RNF20 and facilitates RNF20 degradation through the autophagy-lysosomal pathway (Che et al., 2023). Additionally, RNF20 was identified as a novel host target of 3C-like protease (3Clpro) of severe acute respiratory syndrome coronavirus 2 (SARS-CoV-2), which cleaves RNF20 at a conserved Q521 (Gln521) across species, which subsequently prevents SREBP1 from RNF20-mediated degradation and promotes SARS-CoV-2 replication (Zhang et al., 2021). Besides the aforementioned pathways regulated RNF20 expression levels, RNF20 activity can also be regulated by phosphorylation. In response to DNA double-strand breaks (DSBs) induction in cells, RNF20 can be phosphorylated at Ser172 and Ser553 in an ATM-dependent manner, thus facilitating its activity of damage-induced H2B monoubiquitination (Moyal et al., 2011).

Thirdly, RNF20 activity can also be affected by its co-regulators (Figure 2C). WAC has been identified as a functional partner of RNF20 and is essential for H2B ubiquitination (Zhang and Yu, 2011). WAC interacts with the coiled-coil domain of RNF20 through its C-terminal coiled-coil region and promotes RNF20’s E3 ligase activity for H2B ubiquitination at active transcription sites during gene transcription (Zhang and Yu, 2011), thus regulating multiple biological processes such as cell-cycle checkpoint activation and cell death induction (Zhang and Yu, 2011; Garrido Castro et al., 2018; Meng et al., 2021). Of note, whether the role of WAC on RNF20’s E3 ligase activity is a general function also involved in non-histone protein ubiquitination requires further investigation.

In summary, RNF20 activity is under tight regulation and in a context-dependent manner to enable its precise molecular functions in different tissues.

## 4 The functions of RNF20 in embryonic development

RNF20 has been shown to be broadly expressed in *Xenopus* and mouse since preimplantation stage and constantly expressed in various tissues till adult, including liver, spleen, lung, kidney, heart, skeletal muscle, adipose tissue, oviduct, trachea and brain (Ooga et al., 2015; Robson et al., 2019; Liang et al., 2021). RNF20 is an essential factor for normal embryonic development, which can be demonstrated by preimplantation embryonic lethality of *Rnf20* homozygous knockout mice (*Rnf20* ^*−/−*^) (Tarcic et al., 2016). This is not surprised since RNF20 has been shown to be essential in the regulation of preimplantation development (Ooga et al., 2015; Tarcic et al., 2016; Liu H. et al., 2025). Heterozygous *Rnf20* ± mice are viable and appear normal (Tarcic et al., 2016; Liu H. et al., 2025). Although morphologically normal, *Rnf20* ± mice were shown to be predisposed to acute and chronic colonic inflammation and inflammation-associated colorectal cancer (Tarcic et al., 2016). Moreover, *Rnf20* ± mice were detected of a high incidence of spontaneous lung tumor formation at 1 year of age (Liu H. et al., 2025). Therefore, specific conditional knockout mouse models are helpful for investigating the roles of RNF20 in the development and maintenance of various organs.

Even though RNF20 has been shown to function in multiple tissues such as adipocytes and islet ß-cells, here we mainly focus on the roles of RNF20 in embryogenesis including cardiac and neurodevelopment.

### 4.1 The roles of RNF20 in cardiac development

Heart is the first organ to form during mammalian embryogenesis. The roles of RNF20 in cardiac development have been extensively studied in recent years. First of all, a loss-of-function *RNF20 de novo* mutation (p.Gln83*) was identified in congenital heart disease (CHD) patient in an exome-sequencing based study (Zaidi et al., 2013), implying a role of RNF20 in cardiovascular development. Then Robson et al., revealed a role of rnf20 in cardiac development in *Xenopus*, by showing that knockdown of *rnf20* leads to decreased H2Bub1 marks which are enriched at cilia genes in multiciliated tissue, resulting in abnormal heart looping, defective development of left-right asymmetry, and impaired cilia motility (Robson et al., 2019).

**FIGURE 3:**
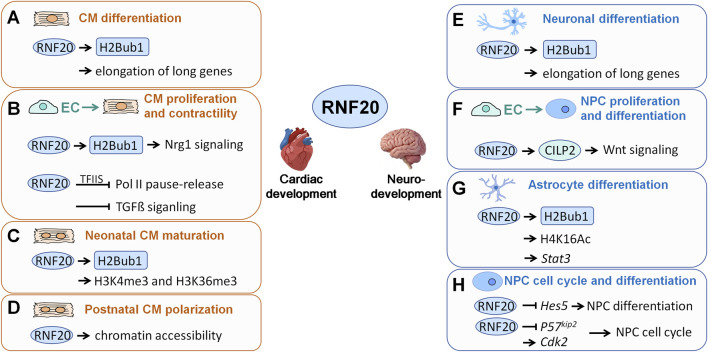
RNF20 functions in cardiac and neurodevelopment. **(A–D)** Roles of RNF20 in cardiac development. **(E–H)** Roles of RNF20 in neurodevelopment. CM: cardiomyocyte; EC: endothelial cells; NPC: neural precursor cell.

Subsequently, several groups investigated the roles of RNF20 in cardiac development further at cellular and molecular levels and with mouse models. Firstly, in cardiomyocyte (CM) differentiation, RNF20 regulates the expression of calcium-signaling and sarcomeric genes through H2Bub1 (Barish et al., 2023). Interestingly, these genes are relatively longer than expected by random chance (Barish et al., 2023). Consistently, H2Bub1 enrichment profile is different on long genes compared with short genes (Barish et al., 2023). H2Bub1 coverage on short genes is over the entire gene body, higher at the 5′ end than the 3′ end of the gene, while on long genes, there is an extra H2Bub1 accumulation peak near the center of the gene, which correlates with amount of full-length transcripts (Barish et al., 2023). These results in hiPSCs-derived CMs are in line with the roles of RNF20-H2Bub1 in stimulating transcriptional elongation and the generation of longer transcripts in an earlier report (Pavri et al., 2006). Together, these data indicate that RNF20 and H2Bub1 are required for CM differentiation by regulating transcriptional elongation of long genes (Figure 3A).

Secondly, in endocardial endothelial cells (EC), on one hand, RNF20 promotes *Nrg1* expression through a RNF20-H2Bub1-dependent mechanism; on the other hand, RNF20 restrains TGF-β signaling by limiting TFIIS recruitment, which is required for the release of RNA polymerase II (Pol II) into active elongation, at TGF-β target genes, thus maintaining endothelial cell identity by inhibiting endothelial-to-mesenchymal transition (EndMT) and regulating CM proliferation and contractility by controlling physiological angiocrine signaling (Dou et al., 2025) (Figure 3B).

Thirdly, in neonatal CMs, RNF20 and RNF40 work through writing of H2Bub1 and exert influences on H3K4me3 and H3K36me3 occupancy at mature sarcomere isoform genes, such as *Myh6* and *Tnni3*, thus regulating the maturational epigenetic landscape and target gene expression during CM maturation (VanDusen et al., 2021) (Figure 3C). This function of RNF20/H2Bub1 is in line with previous reports that H2Bub1 is necessary for H3 methylation and acts as an activating mark for gene expression (Sun and Allis, 2002; Dover et al., 2002; Xiao et al., 2005; Pavri et al., 2006; Kim et al., 2009).

Moreover, polarization of intercalated discs (IDs) is one of the hallmarks of structural maturation of CMs. In postnatal CMs, RNF20 was shown to promote CM polarization through the regulation of genes involved in cell-cell interactions and cytoskeleton organization through regulating the chromatin accessibility landscape in early postnatal heart (Lin et al., 2023) (Figure 3D). Since a significant loss of H2Bub1 was observed in CMs with conditional knockout of RNF20 (Lin et al., 2023) and H2Bub1 has been shown to promote chromatin relaxation (Fierz et al., 2011), the function of RNF20 in CM polarization is most probably through its epigenetic target H2Bub1.

### 4.2 The roles of RNF20 in neurodevelopment

Neurodevelopment is another important process of embryogenesis. By summarizing the roles of RNF20 in neurodevelopment, we found the work pattern of RNF20 in neurodevelopment is similar with that in hiPSC-based CM differentiation aforementioned. Firstly, in ESC-based neuronal differentiation, RNF20 catalyzes H2Bub1 and promotes the transcriptional induction of relatively long genes (Fuchs et al., 2012) (Figure 3E). Secondly, like RNF20 in cardiac endothelial cells that functions in crosstalk with cardiomyocytes by controlling angiocrine signaling, RNF20 in cerebrovascular endothelial cells regulates the expression of secreted protein cartilage intermediate layer protein 2 (CILP2). The endothelium-derived CILP2 alters the downstream cascade signaling of Wnt signaling pathways in neural precursor cells (NPCs) through the interaction with Wnt3a, thus orchestrating the proliferation and differentiation of NPCs by cell-cell communications (Figure 3F). However, how RNF20 regulates CILP2 expression is still unclear and further studies will be needed to clarify this question. Thirdly, RNF20 also affects other histone modifications in neurodevelopment. In astrocytic differentiation, RNF20 functions cooperatively with acetyltransferase MOF to facilitate H4K16ac. RNF20-mediated H2Bub1 cooperating with MOF-mediated H4K16ac activates the transcription of *Stat3*, thus promoting astrocyte production (Figure 3G). In addition, RNF20 also acts as a multifunctional molecule in NPCs. On one hand, RNF20 promotes the expression of Notch signaling effector gene *Hes5*, through the action of *Fezf1* and *Fezf2* genes, and enhanced the differentiation of NPCs (Ishino et al., 2014). On the other hand, *Rnf20* knockdown in NPCs lengthened their cell cycle through the upregulation of *p57* ^*kip2*^ and the downregulation of *Cdk2* (Ishino et al., 2014) (Figure 3H).

Altogether, these reports showed coherent evidence that RNF20 plays important roles in cardiac and neurodevelopment, these molecular modes of actions of RNF20 give a hint that RNF20 may regulate embryonic development in different tissues with some common regulatory mechanisms.

## 5 Mechanisms how RNF20 controls health and diseases

**FIGURE 4:**
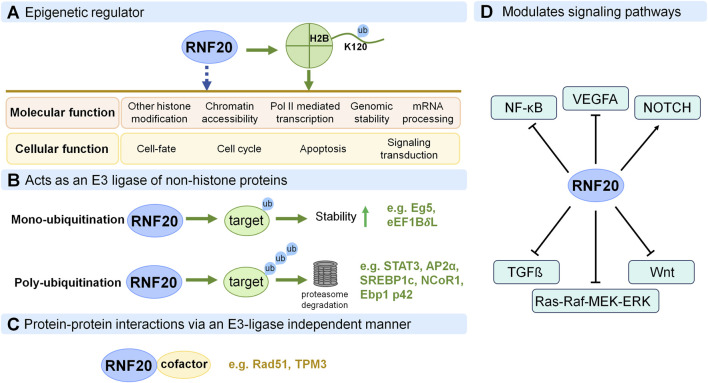
Molecular modes of action of RNF20 in health and diseases. **(A)** RNF20 functions as an epigenetic regulator by monoubiquitinating histone H2B (at K120 in mammalian cells) and exerts effects on other histone modifications, chromatin accessibility, Pol II-mediated transcription, genomic stability and mRNA processing, thus influencing biological processes in different cellular contexts such as cell-fate, cell cycle, apoptosis and signaling transduction. Green single-headed arrow indicates activities triggered by RNF20 via H2Bub1. Blue dashed arrow indicates activities whether RNF20 functions through H2Bub1 or not is still not very clear or independent of H2Bub1 in some cases. **(B)** RNF20 acts as an E3 ligase of non-histone proteins, by monoubiquitination or polyubiquitination. **(C)** RNF20 functions by interacting with cofactors (for example: Rad51 and TPM3) via an E3-ligase independent manner in different contexts. **(D)** RNF20 regulates physiological and pathological processes by modulating signaling pathways.

After the roles of RNF20 in different tissues have been clarified, a question arises that how RNF20 functions in these biological processes. Here we summarize its cellular and molecular modes of actions as follows (Figure 4).

### 5.1 RNF20 functions as an epigenetic regulator

The most well-known function of RNF20 in epigenetic regulation is as the E3 ligase of H2Bub1. In 2003, the Shilatifard and Madhani groups discovered that RNF20 is required for H2Bub1 in yeast cells and most probably is the E3 enzyme that directs Rad6 to ubiquitinate H2B (Wood et al., 2003; Hwang et al., 2003). Subsequently, this conclusion was confirmed in human cells by different groups, suggesting the conserved roles of RNF20 from yeast to humans (Kim et al., 2005; Zhu et al., 2005). RNF20/H2Bub1 was further clarified to function as a transcriptional co-activator on multiple target genes (Bray et al., 2005; Kim et al., 2005; Zhu et al., 2005). Consistently, H2B monoubiquitination by RNF20/RNF40 was shown to be required for active transcription associated histone marks including trimethylation of H3K4, H3K79 and H3K36, as well as for the resolution of H3K4me3/H3K27me3 bivalency on H2Bub1-occupied genes in many different cells (Wood et al., 2003; Hwang et al., 2003; Kim et al., 2005; Kim et al., 2009; Nakanishi et al., 2009; Karpiuk et al., 2012; Xie et al., 2020; VanDusen et al., 2021). Of note, this crosstalk of H2Bub1 with other histone modifications is highly cell-context dependent as implied by that not all of these aforementioned histone marks always show corresponding changes in the same cell types (Kim et al., 2005; Zhu et al., 2005; Hahn et al., 2012; Vethantham et al., 2012). Interestingly, how does RNF20/H2Bub1 regulate other histone modifications? One way could be RNF20/H2Bub1 acts as an upstream regulator for histone modifiers and affect their expression levels, thus regulating target histone modifications (Xie et al., 2017). Another way is RNF20/H2Bub1 could work in concert with histone modifiers to enhance or inhibit their activities (Kim et al., 2009; Jeon et al., 2018). Moreover, RNF20/H2Bub1 could also alter the epigenetic landscape or act as the signal that affects the recruitment of histone modifiers to its target sites (Dover et al., 2002; Wood et al., 2003). It is noteworthy that RNF20/H2Bub1 can regulate the levels of other histone modifications by one or combined aforementioned pathways in different contexts (Liang et al., 2017).

In addition to the effects on other histone modifications, RNF20 also influences the epigenetic landscape by changing the chromatin accessibility (Hooda et al., 2019; Lin et al., 2023). The role of RNF20 in chromatin conformation alteration is likely through H2Bub1, since H2Bub1 level changes are also observed in parallel (Hooda et al., 2019; Lin et al., 2023). Interestingly, loss of RNF20 and H2Bub1 has been shown to be associated with a more open chromatin conformation in fallopian tube epithelial cells (Hooda et al., 2019) while RNF20 promotes the chromatin relaxation in early postnatal heart (Lin et al., 2023). This is not surprising, since H2Bub1 has been shown to be able to either stabilize nucleosomes (Chandrasekharan et al., 2009) or disrupt chromatin compaction (Fierz et al., 2011).

Furthermore, RNF20 and H2Bub1 are found to be tightly associated with Pol II mediated transcription by regulating Pol II pause-release and elongation processes (Pavri et al., 2006; Shema et al., 2011; Fuchs et al., 2012; Barish et al., 2023; Tetik-Elsherbiny et al., 2024; Dou et al., 2025; Liu H. et al., 2025). On one hand, RNF20-mediated H2Bub1 facilitates FACT function, thereby stimulating transcript elongation and the generation of longer transcripts (Pavri et al., 2006). On the other hand, RNF20 hinders the recruitment of transcription elongation factor TFIIS, which is required for the release of RNA Pol II from pausing into active elongation at target genes in some cell types (Shema et al., 2011; Dou et al., 2025).

RNF20/H2Bub1 plays a key role in the regulation not only of transcription but also of genomic stability. DNA damage caused by internal or external damaging agents is a major threat to the integrity of the cellular genome. Defects in double-strand breaks (DSBs) repair can cause genome instability and many human diseases, including cancers. RNF20-mediated H2B monoubiquitination at DSBs plays a critical role in guarding genome stability. Upon DNA damage induction, RNF20 will be recruited to the DNA DSBs sites by mediators such as SUPT16H, a component of FACT and replication protein A (RPA), and phosphorylated in an ATM-dependent manner which mediates the damage-induced H2B monoubiquitylation that is required for optimal DSB repair (Moyal et al., 2011; Oliveira et al., 2014; Liu et al., 2021). RNF20/H2Bub1 regulates chromatin reorganization by promoting H3K4 methylation and SNF2H recruitment at DSBs, facilitating the resection of DNA ends and the recruitment of DNA repair proteins such as BRCA1and RAD51 (Nakamura et al., 2011). Then the DNA damage can be timely repaired by DSB repair pathways including nonhomologous end-joining (NHEJ) and homologous recombination repair (HRR).

Last but not least, RNF20/H2Bub1 can also function in mRNA processing. Transcripts generated by Pol II undergo a precisely orchestrated cascade of processing steps involving 5′ end capping, splicing, 3′ end cleavage and polyadenylation, before being transported to the cytoplasm as export-competent mRNA ribonucleoprotein particles (mRNPs). RNF20/H2Bub1 has been shown to be involved in replication-dependent histone mRNA 3′-end processing and contribute to efficient mRNA nuclear export by controlling the formation of export-competent mRNP (Pirngruber et al., 2009; Vitaliano-Prunier et al., 2012). Recently, RNF20 was reported to regulate the alternative splicing of pro-angiogenic genes, such as *VEGFA* in endothelial cells (Tetik-Elsherbiny et al., 2024). In this context, the authors mentioned that RNF20 could regulate RNA diversity by tightly controlling Pol II pause release at genes involved in the regulation of splicing and/or by the regulation of H2Bub1 levels (Tetik-Elsherbiny et al., 2024). Actually, H2Bub1 is indeed involved in alternative splicing by showing high enrichment at exon-intron boundaries of highly expressed exons and in skipped exons in human cells (Jung et al., 2012), as well as stimulating recruitment of the early splicing factors, namely, U1 and U2 snRNPs, onto nascent RNAs, thus modulating spliceosome assembly and function in budding yeast (Hérissant et al., 2014).

Altogether, all of the present correlative findings reinforce the notion that RNF20 functions in regulation of other histone modifications, chromatin accessibility, Pol II-mediated transcription, genomic stability and mRNA processing, in most of the cases via H2Bub1. In some cases, whether RNF20 functions through H2Bub1 or not is still not very clear or independent of H2Bub1 (Liu H. et al., 2025). Given RNF20/H2Bub1 participates in so many critical molecular processes, it is not surprising that misregulation of RNF20 will cause cellular consequences including cell-fate alteration, disruptions in cell cycle, apoptosis and signaling transduction, leading to various kinds of cancers and congenital disorders.

### 5.2 RNF20 functions as an E3 ligase of non-histone proteins

As we know, proteins can be monoubiquitinated or polyubiquitinated. Monoubiquitinated proteins are usually stable while polyubiquitinated proteins will usually be targeted for degradation by the ubiquitin-proteasome pathway. As an ubiquitin E3 ligase, RNF20 can also function on target proteins for both monoubiquitination and polyubiquitination, beyond H2Bub1. Motor protein Eg5 and heat shock transcription factor eEF1B*δ*L were found to be monoubiquitinated and stabilized by RNF20, thus promoting mitotic spindle assembly and heat shock responsive gene expression (Duan et al., 2016; In et al., 2019). In parallel, RNF20 promotes the polyubiquitination and degradation of STAT3, AP2α, SREBP1c, NCoR1, Ebp1 p42 in multiple cell types (Liu et al., 2009; Lee et al., 2014; Ren et al., 2014; Lee et al., 2017; Jeon et al., 2020; Wang et al., 2023).

### 5.3 RNF20 functions in an E3-ligase-independent manner through interactions with other proteins

RNF20 has been shown to facilitate DNA replication and repair via promoting H2Bub1, which relaxes the closed chromatin structure at DSBs to allow access to repair machinery (Moyal et al., 2011; Nakamura et al., 2011; Northam and Trujillo, 2016; Hung et al., 2017). Interestingly, during homologous recombination (HR), RNF20 can also function as a recombination mediator protein in a manner independent of its E3 ligase activity (Liu et al., 2023). On one hand, RNF20 interacts with Rad51, directs Rad51 to single-strand DNA (ssDNA), and facilitates Rad51-ssDNA filament assembly and strand exchange. On the other hand, RNF20 interacts with the Srs2 to counteract its disrupting effect on the Rad51 filament, thus contribute to HR repair (Liu et al., 2023). The E500, K502 and D484, K486 sites in the coiled coil region of RNF20 are responsible for the interactions between RNF20 and Rad51 and Srs2, respectively. Researchers further found that simultaneous mutation of E500 and K502 residues to alanine (EK2A) completely abolished the interaction between RNF20 and Rad51, but still exhibited normal E3 ligase activity toward H2B, displayed reduced HR survival and repair kinetics (Liu et al., 2023). Moreover, RNF20 RING-domain deleted mutant can also unload Srs2 from ssDNA and partially compensate the DNA damage repair defect, as WT (wild type) RNF20 does, implying an ubiquitin ligase activity independent role of RNF20 in HR (Liu et al., 2023).

Concomitantly, during oocyte meiotic spindle assembly, RNF20 interacts with TPM3 and recruits TPM3 to both centromeres and spindle poles via its coiled-coil motif, which is also not dependent on its E3 ligase activity either (Wang et al., 2024). Researchers showed that RNF20 depletion does not affect H2Bub1 levels in the oocytes (Liu et al., 2023). Moreover, RNF20 RING mutant (RNF20 C922S) of which RNF20’s E3 ligase activity is lost, does not affect its localization on centromeres and spindle poles, and can also rescue the spindle assembly and chromosome alignment defects in RNF20-depleted cells, as WT RNF20 does, suggesting RNF20 functions in oocyte meiotic spindle assembly independent of its E3 ligase activity (Liu et al., 2023).

### 5.4 RNF20 functions through modulating signaling pathways

RNF20 has been shown to regulate gene expression through modulating signaling pathways, including NF-κB, VEGFA, Notch, TGFß, Wnt and Ras-Raf-MEK-ERK cascades (Bray et al., 2005; Tarcic et al., 2016; Zhao et al., 2021; Zhang et al., 2022; Tetik-Elsherbiny et al., 2024; Dou et al., 2025).

#### 5.4.1 NF-κB signaling

As mentioned above, RNF20 heterozygous mice have been shown to be prone to acute and chronic colonic inflammation and inflammation-associated colorectal cancer, due to elevated NF-κB activity. Mechanically, RNF20 inhibits P65 occupancy on chromatin while in favor of P50, thus restricting NF-κB target gene transcription in response to TNF-α (Tarcic et al., 2016). Interestingly, RNF40 acts differently on NF-κB signaling. RNF40 facilitates the expression of NF-κB target genes after TNFα treatment by promotes nuclear translocation of P65 (also called RelA) in colon cancer cells (Kosinsky et al., 2019).

#### 5.4.2 VEGFA signaling

In vessel endothelial cells (ECs), RNF20 regulates alternative splicing of VEGFA (Tetik-Elsherbiny et al., 2024). In control ECs, *Vegfa* coding exons 2–7 were largely excluded, leading to a non-functional transcript, and RNF20 deficiency resulted in the inclusion of exons 2–4/5, leading to the expression of *Vegfa111* isoform in RNF20 depleted ECs, accompanied by elevated VEGFA protein levels (Tetik-Elsherbiny et al., 2024). Elevated VEGFA signaling, followed by activation of the ERK1/2 signaling cascade and ETS family members, plays a central role in activating pro-angiogenic genes and promoting sprouting upon RNF20 loss (Tetik-Elsherbiny et al., 2024).

#### 5.4.3 Notch signaling

The *Drosophila* homolog of RNF20, dBre1, has been shown to be required for the expression of Notch target genes by coupling H2Bub1 to H3K4me3 (Bray et al., 2005). Similarly, mammalian RNF20 also plays a crucial role in regulating Notch target gene expression via interacting with Notch1 intracellular domain (N1ICD) and facilitating N1ICD association to chromatin, as well as H2B monoubiquitination at Notch target genes in vessel ECs, suggesting an evolutionary conserved role of RNF20 in the regulation of Notch-dependent transcriptional program through modulating the histone code (Tetik-Elsherbiny et al., 2024).

#### 5.4.4 TGFß signaling

In embryonic heart, loss of RNF20 led to enhanced TGF-β signaling in ECs (Dou et al., 2025). Mechanically, RNF20 hinders RNA pol II pause release at TGF-β targets by preventing TFIIS binding, thereby inhibiting endothelial-to-mesenchymal transition (EndMT) in ECs, which maintains endothelial identity and contributes to proper heart development and function (Dou et al., 2025).

#### 5.4.5 Wnt signaling

The role of RNF20 in modulating Wnt signaling is indirect. RNF20 deletion in ECs leads a decrease in CILP2-secreted protein (Zhang et al., 2022). CILP2 interacted with Wnt3a and inhibit canonical Wnt signaling, thus influencing the proliferation and differentiation of NPCs and supporting a vascular-to-NPCs crosstalk during brain development (Zhang et al., 2022).

#### 5.4.6 Ras-Raf-MEK-ERK signaling

In porcine preadipocyte differentiation, RNF20 knockdown leads to significant decrease of mRNA levels of *Ras* and *Raf1*, phosphorylated and total protein levels of ERK1/2 and MEK1/2 at either 1 day and/or 2 days after differentiation, implying a role of RNF20 in regulation of Ras-Raf-MEK-ERK cascade in porcine preadipocyte differentiation (Zhao et al., 2021). The detailed molecular mechanisms underlying how RNF20 affects the mRNA or protein levels of components in this cascade still need further investigations.

As functions of RNF20 are highly context-dependent and only a subset of genes is regulated by RNF20 in a specific cell type (Shema et al., 2008; Xie et al., 2017), raising the question that how RNF20 is recruited to its target sites in different cells? One possible way is that RNF20 may interact with specific factors in a context dependent manner. For example, RNF20 physically binds to P53 and is recruited to the promoter of target genes in a P53-dependent manner in cancer cells, where it serves as a transcriptional coactivator of P53 (Kim et al., 2005). In mouse ES cells, RNF20 interacts with Fbxl19, a factor preferentially occupied on CpG island-containing promoters, and is recruited to the promoters of CpG island-containing genes, thus maintaining normal differentiation of mouse ES cells (Lee et al., 2017). Moreover, MED23 has also been shown to associate with RNF20/40 complex, which is critical for the recruitment of RNF20/40 to chromatin, and regulates H2Bub1 on a subset of MED23-controlled genes (Yao et al., 2015). Another possible way may be the chromatin context target genes located, builds a specific microenvironment that affects the binding of RNF20 (Xie et al., 2017). This is also consistent with the observation that not all of the histone markers known affected by RNF20/H2Bub1 always show corresponding changes in a specific cell type.

Taken together, RNF20 functions as a multifunctional factor in different biological processes. Given that the biological regulation is very complicated, RNF20 could act in one or more manners in parallel in different contexts. The cofactors of RNF20 and its interplay with the surrounding chromatin may help to determine which ways to work.

## 6 Misregulation of RNF20 results in cancers and developmental disorders in humans

**FIGURE 5:**
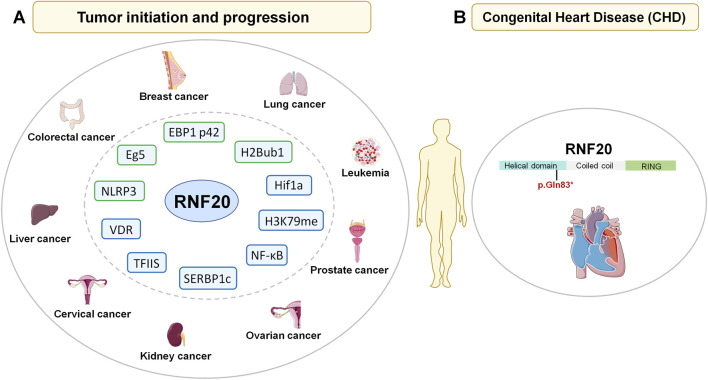
Misregulation of RNF20 results in cancers and congenital heart disease in humans. **(A)** RNF20 is associated with tumor initiation and progression of multiple cancers. The downstream effectors of RNF20 are listed. Factors regulated by RNF20 via mono- or poly-ubiquitination are shown in green boxes. Factors with its level or activity regulated by RNF20 by other pathways or unknown are shown in blue boxes. **(B)** RNF20 mutation has been found in patients with congenital heart disease (CHD). Reported CHD associated *RNF20* mutation (p.Gln83*) is highlighted.

Ubiquitously expressed and functions in multiple tissues, it comes as no surprise that RNF20 misregulation will be associated with human diseases. Indeed, mutations in RNF20 have been found in a variety of cancers according to the cBioPortal for cancer genomics database (Zhao et al., 2023), as well as in developmental disorders such as congenital heart diseases (Zaidi et al., 2013). In line with this, a growing number of studies have showed critical roles of RNF20 in tumor initiation and progression of many cancer types including cervical cancer, breast cancer, colorectal cancer, leukemia, prostate cancer, ovarian cancer, kidney cancer, lung cancer and liver cancer (Jaaskelainen et al., 2012; Wang et al., 2013; Dickson et al., 2016; Duan et al., 2016; Lee et al., 2017; Tarcic et al., 2017; Zhang et al., 2017; Garrido Castro et al., 2018; Hooda et al., 2019; Wang et al., 2020; Liu et al., 2024a; Fatima et al., 2024; Jansari et al., 2025; Liu H. et al., 2025) (Figure 5). However, the roles of RNF20 in different cancers vary greatly.

### 6.1 RNF20 in cervical cancer

In 2008, Shema et al. showed that RNF20 selectively represses the expression of a subset of proto-oncogenes in HeLa cells which are highly enriched by H2Bub1, and acts as a putative tumor suppressor (Shema et al., 2008). They further illustrated that RNF20 exerts this suppressing effect on this subset of RNF20-suppressed genes by interfering with chromatin recruitment of TFIIS, a factor capable of relieving stalled RNA pol II, and inhibiting the interaction between TFIIS and the PAF1 complex, thus hindering transcriptional elongation (Shema et al., 2011). Of note, this subset of RNF20-suppressive genes is substantially overlapped with the group of genes induced by epidermal growth factor (EGF) (Shema et al., 2008). This result is in line with the reported conclusion that stimulus-induced genes, which presumably require rapid changes in chromatin structure to become active, appear to particularly require H2Bub1 to facilitate recruitment of the FACT histone chaperone complex and induce dynamic changes in chromatin structure (Johnsen, 2012). This could probably explain the observation that genes suppressed by RNF20 are associated with closed chromatin but higher constitutive RNA Pol II occupancy, highly enriched H2Bub1 and associated histone modifications such as H3K4 methylation and H3 acetylation which are already well in place and can be poised for rapid activation by environmental cues (Shema et al., 2008).

Whereas, a report published recently showed that RNF20/RNF40/H2Bub1-axis mediates increased peroxisome functions and consequently stimulates aggressiveness in cervical cancer cells including HeLa cells through resistance to ferroptosis, therefore, playing a tumor-supportive role in cervical cancer (Jansari et al., 2025). Researchers showed that loss of RNF20 and RNF40 leads to downregulation of peroxisome-related genes and thus impaired peroxisomal biogenesis, ROS metabolism, and increased lipid peroxidation, ultimately resulting in ferroptotic programmed cell death induction (Jansari et al., 2025). Compared with the putative tumor suppressor roles of RNF20 reported in HeLa cells which focused on RNF20-suppressive and EGF induced genes, in this report, RNF20/RNF40/H2Bub1 activated genes including *PRDX5*, *PEX6*, and *PMVK* are supposed to be the key targets that function for its tumor-supportive roles, implying that RNF20 may play multiple roles even in the same cell type to respond to different environmental stimulatory signals.

### 6.2 RNF20 in breast cancer

Unlike the Seemingly contradictory roles of RNF20/RNF40 in cervical cancer, the actions of RNF20 in breast cancers are quite clear that RNF20 plays a tumor suppressive role in the basal-like breast cancer cells and tumor promoting role in luminal breast cancer cells (Tarcic et al., 2017). In line with this, RNF20/40 complex was found to monoubiquitinate and stabilize motor protein Eg5, high expression of which is associated with poorer overall survival of luminal breast cancer patients, and participates in spindle assembly during mitosis, thus promoting breast carcinogenesis (Duan et al., 2016). RNF20 was also shown to inhibit the tumor suppressive activity of Ebp1 p42 (one of the isoforms encoded by *PA2G4* gene) through mediating its polyubiquitination and degradation in breast cancer cells (Liu et al., 2009). In addition, RNF20/40 and histone chaperone FACT have been shown to cooperate with hypoxia-inducible factor 1 (HIF1) to activate transcription of target genes in human breast cancer cells in response to hypoxia (Lyu et al., 2024).

### 6.3 RNF20 in colorectal cancer

Loss of RNF20/RNF40/H2Bub1 has been shown to promote intestinal inflammation via decreased Vitamin D Receptor (VDR) activity, thus playing critical roles in inflammatory bowel disease (IBD) (Kosinsky et al., 2021). This is in line with the report that downregulation of RNF20 and H2Bub1 promotes chronic colonic inflammation and inflammation-associated colorectal cancer (Tarcic et al., 2016). Mechanically, RNF20 depletion, leading to decreased H2Bub1, augments the ability of the proinflammatory cytokine tumor necrosis factor alpha (TNF-α) to upregulate a panel of inflammation-associated genes through elevated NF-κB activity in *Rnf20* ± mice (Tarcic et al., 2016). Consistently, human data also showed lower levels of RNF20/RNF40/H2Bub1 in colon adenocarcinoma, implicated a role of RNF20 in colonic tumor suppression (Tarcic et al., 2016). However, studies from other groups showed that RNF40 plays a central role in the maintenance of tumorigenic features and inflammatory signaling by promoting nuclear NF-κB activity and controlling the expression of genes encoding central apoptotic regulators (Kosinsky et al., 2019; Schneider et al., 2019). Like the role of RNF20 in breast cancer, Fatima et al. showed that RNF20 also target mitotic kinesin Eg5 for monoubiquitination in colon cancer cells (Fatima et al., 2024). They also identified Albendazole, an anti-helminth drug, showing strong inhibitory effects on the tumorigenic potentials of colon cancer cells by modulating the expression of RNF20, thereby leading to cell cycle arrest and apoptosis (Fatima et al., 2024). Albendazole also enhances chemosensitivity of colon cancer cells to 5-fluorouracil (5-FU), providing evidence that repurposing albendazole for colon cancer treatment (Fatima et al., 2024).

In these reports, the roles of RNF20 in colorectal cancer are seemingly conflicting. One possible reason is that RNF20 have multiple functions. Loss of RNF20 in inflammation-associated colorectal cancer also showed decreased H2Bub1 level, implying that RNF20 could function through H2Bub1 in this process (Tarcic et al., 2016). While RNF20 can not only monoubiquitinate H2B, but also has been found to monoubiquitinate Eg5, thus regulating the cell cycle and apoptosis in colon cancer cells (Fatima et al., 2024). Another key point is the seemingly inconsistent results from human tissue data analysis could be due to the differences of tumor stages as well as subtypes of cancer. In addition, the functions of RNF20 and RNF40 could be different in colorectal cancers. RNF20 and RNF40 not only can work together to monoubiquitinate H2B, but also have their own non-histone substrates, as shown in different contexts (Chin et al., 2002; Liu et al., 2024b; You et al., 2025; Huang et al., 2026). The observations that the H2Bub1 is distributed independently from RNF40 in colorectal cancer samples support this notion (Kosinsky et al., 2019).

### 6.4 RNF20 in MLL-rearranged acute leukaemia

MLL-rearranged acute leukemia (ALL) is a subtype of leukemia, characterized by hyperleukocytosis, aggressive behavior with early relapse, relatively high incidence of central nervous system (CNS) involvement, and poor prognosis (El Chaer et al., 2020). RNF20 was identified to play a supporting role in the pathogenesis of MLL-fusion leukemia (Wang et al., 2013). Mechanically, RNF20/H2Bub1 functions to maintain local DOT1L-mediated H3K79 methylation at MLL-AF9 target genes (Wang et al., 2013). Therefore, suppression of RNF20 leads to impaired leukemia progression associated with reduced expression of MLL-AF9 target genes (Wang et al., 2013). Intriguingly, histone deacetylase inhibitor (HDACi) Panobinostat (LHB589) was identified to target RNF20/RNF40/WAC-H2Bub1 axis in MLL-rearranged ALL cells (Garrido Castro et al., 2018). LHB589 monotherapy in a xenograft mouse model showed strong anti-leukemic effects, extending survival and reducing overall disease burden (Garrido Castro et al., 2018). This study suggests that LBH589 can exert cross-inhibition of different epigenetic pathways, with a direct link between HDAC inhibition and the downregulation of RNF20 activity in LBH589-sensitive ALL cells (Garrido Castro et al., 2018).

### 6.5 RNF20 in prostate cancer

In metastatic prostate cancer, RNF20 expression is decreased (Jaaskelainen et al., 2012). RNF20 was shown to interact with androgen receptor (AR) and stimulate its transcriptional activity in prostate cancer cells (Jaaskelainen et al., 2012). Silencing of RNF20 in prostate cancer cells results in a general decrease in global H2Bub1 level, and leads to gene-selective effects on AR target expression (Jaaskelainen et al., 2012). Depletion of RNF20 retards the growth of prostate cancer cells by downregulation of cell cycle associated genes including *MYC*, *MKI67* and *GTSE1* (Jaaskelainen et al., 2012).

### 6.6 RNF20 in ovarian cancer

Through a large cohort of high-grade serous ovarian cancers (HGSOC) analysis, 77% (313 of 407) of tumors showed loss of global H2Bub1 level, which was seen at all stages (I-IV) of HGSOC, indicating it is a relatively early epigenomic event in this aggressive malignancy (Dickson et al., 2016). In line with this, Hooda et al. reported that heterozygous loss of RNF20 defines the majority of HGSOC tumors through the analysis of a cohort of 579 HGSOC cases from The Cancer Genome Atlas (TCGA) database (Hooda et al., 2019). H2Bub1 level is also lost or downregulated in a large proportion of invasive HGSOC tumors, implicating RNF20/H2Bub1 loss as an early event in the development of serous ovarian carcinoma (Hooda et al., 2019). RNF20 and H2Bub1 depletion escalates oncogenic behavior in cells derived from late-stage HGSOC (Hooda et al., 2019). Mechanically, ATAC-seq and RNA-seq results revealed that loss of RNF20/H2Bub1 leads to a more accessible chromatin state that causes upregulation of immune modulators that stimulate growth and migration of early HGSOC precursors (Hooda et al., 2019).

### 6.7 RNF20 in kidney cancer

Clear cell renal cell carcinoma (ccRCC) is the most common subtype of kidney cancers, characterized by ectopic lipid accumulation (Lee et al., 2017). Lee et al. reported that RNF20 mRNA expression is downregulated in ccRCC tumors compared to that in patient-matched normal kidney tissues (Lee et al., 2017). Moreover, low RNF20 expression is closely associated with poor survival in ccRCC patients, implying that RNF20 acts as a tumor suppressor in ccRCC (Lee et al., 2017). They further found that RNF20 hindered lipogenesis and cell proliferation by inhibiting sterol regulatory element-binding protein 1c (SREBP1c). SREBP1c further regulates cell cycle progression by inducing the expression of its target gene pituitary tumor-transforming gene 1 (*PTTG1*) in ccRCC cells (Lee et al., 2017).

### 6.8 RNF20 in lung cancer

Lung cancer is the leading cause of cancer-related death worldwide (Leiter et al., 2023). It is broadly categorized into small-cell lung cancer and non-small-cell lung cancer (NSCLC); NSCLC comprises >85% of all cases and can be further classified by histological subtype (Leiter et al., 2023). Globally, the most common histological subtype of NSCLC is lung adenocarcinoma (LUAD) (40%), followed by squamous cell carcinoma (25%) (Leiter et al., 2023).

RNF20/H2Bub1 levels are significantly reduced in LUAD tissues and loss of RNF20/H2Bub1 is significantly correlated with enhanced malignancy and promote disease progression in LUAD, suggesting RNF20 and H2Bub1 act as tumor suppressor in LUAD (Zhang et al., 2017). Very recently, Liu et al. demonstrated the detailed roles of RNF20 in lung cancer (Liu H. et al., 2025). They showed that ablation of a single *Rnf20* allele significantly increases the incidence of lung tumors in mice (Liu H. et al., 2025). Mechanically, on one hand, *Rnf20* haploinsufficiency results in inadequate tumor suppression via the RNF20-H2Bub1-p53 axis; one the other hand, *Rnf20* haploinsufficiency induces DNA damage, cell growth, epithelial-mesenchymal transition (EMT) and metabolic rewiring through HIF1α-mediated RNA pol II promoter-proximal pause release, which is independent of H2Bub1 (Liu H. et al., 2025). In contrast to the necessary role of RNF20/H2Bub1 for HIF1 transcriptional activity in breast cancer cells, RNF20 acts to decrease HIF1α level and activity by controlling the expression of RBX1 in lung cancer cells (Liu H. et al., 2025).

### 6.9 RNF20 in liver cancer

RNF20 has been shown to inhibit the progression of liver fibrosis via H2Bub1 (Chen et al., 2021). Liver fibrosis is a chronic liver injury that leads to liver cirrhosis and liver cancer. In line with this, Liu et al. found that over-expression of RNF20 hinders cell proliferation, metastasis and Warburg effect of liver cancer cells by promoting NLRP3 ubiquitination, implying a tumor suppressing role of RNF20 in liver cancer (Liu et al., 2024a).

In summary, RNF20 plays multiple roles in different cancers in which its molecular functions appear to be complicated and likely depends both upon tumor stages as well as the types of malignancy. Interestingly, the presence or absence of RNF20 in different cell types or even in the same cell type facing different stimulatory signals, may serve to facilitate or repress gene expression in a context-dependent manner which is still not completely understood so far.

### 6.10 RNF20 in human congenital disorders

In addition to mutations of RNF20 found in a variety of cancers, a nonsense mutation of RNF20 (p.Gln83*; highlighted in Figure 5) has been found in congenital heart disease (CHD) patient through an exome sequencing based study (Zaidi et al., 2013). This loss-of-function mutation leads to complete loss of the coiled-coil and RING domains of RNF20. Furthermore, bulk RNA-sequencing data revealed reduced RNF20 expression in Tetralogy of Fallot (ToF) patients compared to control donors (Dou et al., 2025). Importantly, studies from different groups have unraveled the key roles of RNF20 in heart development in multiple models (Robson et al., 2019; Barish et al., 2023; Dou et al., 2025).

Interestingly, another report pointed that newborns with CHD have a high risk of neurodevelopmental disabilities, particularly in cases associated with mutated genes highly expressed in both heart and brain (Homsy et al., 2015). RNF20 could be such a gene, even though there is no RNF20 mutations found in neurodevelopmental disorders so far. RNF20 expresses highly in both developing heart and brain, and has been shown to be essential for both heart and brain development as mentioned above. The research of RNF20 in different organs surely will provide proposed mechanisms of pathogenesis of human diseases.

## 7 Conclusion

RNF20 is a pivotal E3 ligase that plays critical roles in multiple physiological and pathological processes. Early studies have revealed its important roles in monoubiquitination of histone H2B. In recent years, a growing number of researches have revealed a more complex regulatory landscape, with not only the downstream regulatory pathways of RNF20, but also upstream controls of RNF20 activity. In this review, we give a comprehensive summary on the current knowledge of RNF20 in embryonic development and RNF20 misregulation associated human diseases; decode its upstream and downstream regulatory patterns to provide molecular basis for associated human diseases.

Besides, E3 ligases confer specificity to ubiquitination when it transfers ubiquitin from an E2 to a particular substrate, thus they are attractive candidates as drug targets (Cohen and Tcherpakov, 2010). Currently, significant progress has been achieved in ubiquitin–proteasome system-dependent targeted protein degradation using strategies like proteolysis-targeting chimeras (PROTACs) (Liu P. et al., 2025; Zhang et al., 2025). As an E3 ligase, one promising area for future investigation for RNF20 is the identification and development of its potential as a drug target. In addition to developing new drugs targeting RNF20, identifying new functions of existing drugs is another direction. More importantly, identification of the inhibitory roles of LBH589 (Panobinostat, Farydak®) in leukemia and Albendazole in colon cancer cells on RNF20 is surely an exciting advance in this area (Garrido Castro et al., 2018; Fatima et al., 2024). Researchers showed that monotherapy with LBH589 exerts strong *in vivo* efficacy against human MLL-rearranged ALL in xenotransplanted mice (Garrido Castro et al., 2018). Moreover, this antineoplastic effect of LBH589 is confirmed also in ALL with t(4; 11) (Moreno et al., 2022). However, antagonistic effects will be induced when it is used in combination with MTX or 6MP (Moreno et al., 2022). Considering that LBH589 is a pan-inhibitor of HDACs, theoretically it could interfere with the activity of enzymes that control the intracellular metabolism of MTX and 6MP, reducing the activity of these chemotherapeutics, resulting in antagonistic effects on these combinations (Moreno et al., 2022). Albendazole treatment in colon cancer can efficiently reduce RNF20 expression, leading to the inhibition of proliferation and other characteristics of aggressive cancer in colon cancer cell lines, mouse-derived organoids, xenograft/syngeneic models, chemoresistant colon cancer cells, and colon cancer stem cells (Fatima et al., 2024). Furthermore, albendazole has also been shown significantly increases the efficacy of traditional chemotherapeutics and can trigger cytotoxicity in 5-FU- and oxaliplatin-resistant colon cancer cells as well as colon cancer stem cells (Fatima et al., 2024). Although these studies showed promising results, they are only preliminary findings based on laboratory and animal models. No large-scale human trials have confirmed the safety and efficacy of them as cancer treatment drugs so far. Further research, including clinical trials, is needed before they can be used in standard cancer therapy.

While numerous studies have showed changes of RNF20 levels are detected in multiple cancers, RNF20 to be exploited for tumor therapy still faces great challenges. First of all, RNF20 is a multifunctional factor and its roles in different contexts of carcinogenesis and metastasis are currently not fully understood. In order to exploit RNF20 as a potential therapeutic target, it will probably first be necessary to determine the exact roles of RNF20 in different types or subtypes of cancers and at different tumor stages. This also raises the second challenge, that how to target RNF20 in the specific cancer cells since RNF20 is ubiquitously expressed in multiple tissues. Thirdly, the side-effects of the drugs targeting RNF20 should also be paid more attention and therefore more in-depth exploration and more adequate validation are needed to elucidate the clinical value of RNF20 in cancers.

Even though extensive studies continue to reveal new insights into the function of RNF20 recently, our current understanding of RNF20 is still far from enough. RNF20/RNF40-H2Bub1 machinery has been shown to regulate the expression of specific subset of genes in a highly context-dependent manner (Shema et al., 2008; Xie et al., 2017). Future investigations elucidating the full activities of RNF20 in different cellular environments are warranted and will lay foundation for clinical implications regarding RNF20 dysfunction associated human diseases and will also speed up the drug discovery process.
